# Prevalence of thyroid autoantibodies in patients with systematic autoimmune rheumatic diseases. Cross-sectional study

**DOI:** 10.1590/1516-3180.2017.0089110617

**Published:** 2017-09-28

**Authors:** Rayana Taques Posselt, Vinícius Nicolelli Coelho, Danieli Cristina Pigozzo, Marcela Idalia Guerrer, Marília da Cruz Fagundes, Renato Nisihara, Thelma Larocca Skare

**Affiliations:** I Medical Student, Medicine Department, Faculdade Evangélica do Paraná, Curitiba (PR), Brazil.; II MD. Attending Physician, Medicine Department, Faculdade Evangélica do Paraná, Curitiba (PR), Brazil.; III PhD. Professor, Medicine Department, Faculdade Evangélica do Paraná and Universidade Positivo, Curitiba (PR), Brazil.; IV PhD. Rheumatologist and Professor, Medicine Department, Rheumatology Unit, Hospital Universitário Evangélico de Curitiba (HUEC), Curitiba (PR), Brazil.

**Keywords:** Thyroid, Autoimmunity, Rheumatic disease.

## Abstract

**BACKGROUND:**

Thyroid autoimmunity is more common in patients with rheumatic diseases than in healthy populations. The degree of association seems subject to influence from patients’ geographical location. Here, we aimed to ascertain the prevalence of thyroid autoantibodies in a cohort of patients with systemic rheumatic disease and the degree of association between its presence and inflammatory activity.

**DESIGN AND SETTING:**

Cross-sectional observational study in a rheumatology unit.

**METHODS:**

301 patients with systemic lupus erythematosus (SLE), 210 with rheumatoid arthritis (RA), 58 with scleroderma (SSc) and 80 with spondyloarthritis (SpA) were studied regarding thyroid function (TSH and T4), anti-thyroglobulin (TgAb) and anti-thyroperoxidase (TPOab) and compared with 141 healthy controls. Disease activity in patients with rheumatic disease was assessed through appropriate indexes.

**RESULTS:**

There were more antithyroid antibodies in SLE patients with hypothyroidism (P = 0.01; odds ratio, OR 2.7; 95% confidence interval, CI: 1.20-6.26) and in those without hypothyroidism (P = 0.06; OR 2.4; 95% CI: 1.28-4.55) than in controls. SSc patients also showed: P = 0.03 both with antithyroid antibodies and hypothyroidism (OR 3.4; 95% CI: 1.06-10.80) and without hypothyroidism (OR 3.1; 95% CI: 1.11-0.13). RA and SpA patients had the same prevalence as controls (P not significant). Presence of autoantibodies with and without hypothyroidism was not associated with the activity or functional indexes evaluated.

**CONCLUSION:**

SLE and SSc were associated with higher prevalence of thyroid autoantibodies in patients with and without hypothyroidism, unlike SpA and RA. There was no link between thyroid autoantibody presence and disease activity or functional impairment.

## INTRODUCTION

Autoantibodies against the thyroid seem to be more common in patients with rheumatic diseases than in the normal population.^1^^,^^2^ Their appearance may be linked to an associated autoimmune thyroid disease, although sometimes they lack a clear clinical meaning. The major antigens driving the appearance of thyroid autoantibodies are thyroglobulin (Tg), thyroid peroxidase (TPO) and thyroid-stimulating hormone receptor (TSH-R).^3^^,^^4^

Tg is a protein from which the thyroid hormones (T3 and T4) are produced. It has four to six epitopes that are believed to be recognized by B cells that are involved in the autoantibody response. TPO, formerly known as microsomal antigen, is an enzyme that catalyzes iodination of the tyrosine residues of thyroglobulin to form monoiodotyrosine and diiodotyrosine.^5^ TPO is found on the apical surface and cytoplasm of thyroid follicular cells.^4^ Unlike Tg, TPO is capable of inducing antibody-dependent cell-mediated cytotoxicity and is considered to be more specific for autoimmune thyroid disease.^3^^,^^4^

The most common autoimmune thyroid disease is Hashimoto thyroiditis, which is characterized by gradual thyroid failure with or without goiter development.^3^ Nearly all Hashimoto thyroiditis patients have high serum concentrations of antibodies against one or more thyroid antigens that are produced by lymphocytic infiltrate in the thyroid gland or, to a small extent, by regional lymph nodes or bone marrow.^4^

Hypothyroidism and rheumatic diseases share common clinical findings such as arthralgias, arthritis, myalgias, myopathy and fatigue,^6^^,^^7^^,^^8^ which need to be correctly diagnosed to be properly treated. Knowing the degree of association between rheumatic and thyroid diseases may help the clinician to yield the correct decision. Systemic lupus erythematosus (SLE),^9^^,^^10^ rheumatoid arthritis (RA),^11^^-^^12^ scleroderma (SSc),^13^^,^^14^ Sjögren’s syndrome^15^ and spondyloarthritis (SpA)^16^ have been associated with increased presence of Hashimoto thyroiditis and/or thyroid autoantibodies. However, the degree of such associations seems to be different according to the sample studied, given that both rheumatic and thyroid diseases are influenced by genetic background and exposure to environment triggers. Moreover, iodine intake is another variable to be taken into account.^5^^,^^17^ Also, in some of the rheumatic diseases mentioned, such as SpA and SSc, studies in this context are scarce.

In the present study, we aimed to estimate the prevalence of thyroid autoantibodies among patients with systemic rheumatic disease (SLE, RA, SSc and SpA) and its association with disease activity and functional impairment.

## METHODS

This was a cross-sectional observational study with a convenience sample, conducted in a single rheumatology unit. The study had previously been approved by the local Research Ethics Committee. All the individuals included signed a consent statement.

Participants in this study presented rheumatic disease in which the onset was after they had reached 16 years of age. To be included, SLE patients needed to fulfill at least four of the classification criteria for SLE published by the American College of Rheumatology (ACR) in 1997.^18^ RA patients needed to have at least four of the 1987 classification criteria from the ACR.^19^ SSc patients needed to achieve at least nine points in the ACR/2013 (EULAR) classification criteria.^20^ SpA patients needed to fulfill the criteria of the Assessment of SpondyloArthritis International Society (ASAS).^21^

All patients were recruited in a single rheumatology unit according to arrival order for routine consultations and willingness to participate in the study, during a two-year period.

The patients’ medical files were reviewed for demographic, clinical and treatment data. Blood samples were collected to determine the TSH, T4 and antithyroid antibody levels. The reference values for TSH ranged from 0.4 to 5.0 mU/l and free T4 from 4.5 to 12.0 μg/dl, and both were measured by means of chemiluminescence (Architect, Abbott). Patients were considered to have hypothyroidism when TSH was > 5.0 mU/ml and free T4 < 4.5 μg/dl or when they were using thyroid replacement therapy. Serum autoantibody levels for Tg and TPO were determined by means of immunometric assays (INOVA-KIT, Quanta Lite, Inova Diagnostics, San Diego, CA, USA). Test results were considered positive if the levels were > 50 IU/ml for TPOab (antithyroid peroxidase antibodies) and > 325 IU/ml for TgAb (antithyroglobulin antibody), as specified by the manufacturer.

At the time of blood collection, the SLE patients’ disease activity and cumulative damage index was determined through the SLE disease activity index (SEDAI)^22^ and the Systemic Lupus International Collaborating Clinics/American College of Rheumatology damage index (SLIIC/ACR),^23^ respectively. For RA patients, the Disease Activity Score 28 using Erythrocyte Sedimentation Rate (DAS28-ESR)^24^ and the Health Assessment Questionnaire (HAQ)^25^ were used to quantify disease activity and the corresponding functional status. For SpA patients, data from the Bath Ankylosing Spondylitis Disease Activity Index (BASDAI)^21^ and the Bath Ankylosing Spondylitis Functional Index (BASFI)^21^ were gathered. For SSc patients, the Rodnan modified index (Rodnan-m)^26^ was measured to evaluate the degree of skin involvement, and the Medsger index^27^ to evaluate the degree of systemic involvement.

Patients from gynecology and ophthalmology units, without known inflammatory or autoimmune disease, were included as controls. They were included according to the order in which they arrived for consultations and according to whether they were willing to participate in the study. To be included, these individuals needed to live in the same geographical area as the patients. The patients and controls were from the same hospital, which only attends patients through the Brazilian National Health System.

The data were gathered into frequency and contingency tables. Rheumatic disease patients with thyroid autoantibodies were evaluated in two main groups: those with anti-TPO antibodies and hypothyroidism (who possibly presented Hashimoto thyroiditis) and those with antibodies without thyroid dysfunction (who possibly presented polyclonal activation of B cells). Logistic regression models were built to study associations between rheumatic diseases and (a) positivity for anti-TPO antibodies with hypothyroidism and (b) positivity for antithyroid antibodies without hypothyroidism (individuals who were negative for antithyroid antibodies were the reference group). The models were adjusted for age, gender and tobacco exposure, since these might affect the prevalence of thyroid autoimmunity.^17^ Positivity for or absence of thyroid autoantibodies was used as the dependent variable. Comparisons of activity and harm indexes for rheumatic diseases according to the presence of antithyroid antibodies were made using the unpaired t test, or the Mann-Whitney test for pairs of samples, or one-way analysis of variance (ANOVA) and the Kruskal-Wallis test for groups of three samples, according to data distribution. The significance level used was 5%.

## RESULTS

As shown in Table 1, a total of 649 individuals with rheumatic diseases were included: 301 patients with SLE; 210 with RA (61% seropositive); 58 patients with SSc (62% limited, 32.8% diffuse form and 5.2% sine scleroderma); and 80 patients with SpA (66.2% with ankylosing spondylitis, 11.2% with psoriatic arthritis and 22.5% with other forms). Over the study period, 141 controls were included. This Table also demonstrates that SLE and SSc patients had higher prevalence of antithyroid antibodies, either with or without hypothyroidism.

**Table 1:** Epidemiological data and prevalence of antithyroid antibodies (ATA) in rheumatic disease patients (n = 649)

	SLE N = 301	RA N = 210	SSc N = 58	SpA N = 80	Controls N = 141
Female gender	282 (93.6%)	191(90.9%)	55 (94.8%)	33 (41.2%)	130 (92.1%)
Median age in years (with IQR)	42 (31-51)	53 (47-61)	56 (39-61)	49 (44-57)	39 (28.5-52)
Hypothyroidism	49 (16.2%)	34 (16.1%)	16 (27.5%)	12 (15%)	16 (11.3%)
At least one ATA	99 (32.8%)	41 (19.5%)	21 (36.2%)	12 (15 %)	23 (16.3%)
Anti-TPO with hypothyroidism	38 (12.6%)	24 (11.4%)	12 (20.6%)	9 (11.2%)	8 (5.6%)
Anti-TPO without hypothyroidism	12 (4.3%)	8 (3.8%)	3 (5.1%)	0	12 (8.5%)
Anti-Tg without hypothyroidism	43 (14.2%)	8 (3.8%)	3 (5.1%)	3 (3.7%)	7 (4.9%)

N = number; IQR = interquartile rate; SLE = systemic lupus erythematosus; RA = rheumatoid arthritis; SSc = scleroderma; SpA = spondyloarthritis. TPO = thyroid peroxidase; Tg = thyroglobulin.

Table 2 shows a comparison of prevalences of presence of TPO autoantibodies with hypothyroidism (presumably Hashimoto thyroiditis) and presence of autoantibodies (TPO and/or Tg) without hypothyroidism (most likely with polyclonal activation of B cell) among rheumatic disease patients, in relation to controls. In this Table, it can be seen that SLE and SSc patients (but not SpA and RA patients) had higher prevalence of antithyroid autoantibodies than that of controls. Table 3 shows that there was no association between presence of thyroid antibodies and the functional and/or activity indexes of these diseases.

**Table 2:** Comparison of the prevalence of hypothyroidism plus TPO antibodies and the prevalence of positivity for thyroid autoantibodies without hypothyroidism, between patients with rheumatic diseases and controls*

	Anti-TPO with hypothyroidism	Anti-TPO and/or anti-Tg without hypothyroidism
Systemic lupus erythematosus	OR = 2.7 (1.20-6.26); P = 0.01	OR = 2.4 (1.28-4.55); P = 0.06
Rheumatoid arthritis	OR = 1.7 (0.62-4.66); P = 0.29	OR = 1.3 (0.56-3.00); P = 0.53
Scleroderma	OR = 3.4 (1.06-10.80); P = 0.03	OR = 3.1 (1.11-0.13); P = 0.03
Spondyloarthritis	OR = 1.1 (0.88-1.02); P = 0.99	OR = 0.49 (0.05-4.05); P = 0.50

*corrected according to age, gender and tobacco exposure. Individuals who were negative for antithyroid antibodies were the reference group. OR = odds ratio, with 95% confidence interval in parentheses. TPO = thyroid peroxidase; Tg = thyroglobulin.

**Table 3:** Comparison of activity and functional indexes for rheumatic diseases in patients with and without antithyroid autoantibodies (ATA)

		Total Sample	Anti-TPO with hypothyroidism	ATA without hypothyroidism	Without ATA	P
SLE	SLEDAI (median [IQR])	0 [0-2.0]	0 [0-2.0]	0 [0-1.5]	0 [0-2.0]	0.45
	SLICC/ACR (median [IQR])	2.0 [1.0-4.0]	2.0 [1.0-3.0]	2 [1.0-4.0]	2 [1.0-4.0]	0.89
RA	DAS28-ESR (mean ± SD)	3.7 ± 1.3	4.2 ± 1.6	3.6 ± 1.1	3.6 ± 1.3	0.18
	HAQ (median [IQR])	1.1 [0.6-1.6]	1.2 [0.6-2.0]	1.0 [0.5-1.1]	1.1 [0.5-1.6]	0.72
SSc	Rodnan-m	15.0 ± 10.1	11.1 ± 9.8	15.3 ± 7.4	15.8 ± 10.8	0.70
	Medsger index (median [IQR])	5.0 [4.0-7.7]	5.5 [3.0-9.0]	4 [3.0-7.0]	6 [4.0-8.0]	0.43
SpA	BASDAI (mean ± SD)	3.8 ± 2.4	4.8 ± 2.6	*	3.7 ± 2.3	0.20
	BASFI (mean ± SD)	4.2 ± 2.7	5.4 ± 2.8	*	4.0 ± 2.7	0.17

*number too low. SLE = systemic lupus erythematosus; SLEDAI (SLE disease activity index); SLIIC/ACR = Systemic Lupus International Collaborating Clinics/American College of Rheumatology Damage Index; RA = rheumatoid arthritis; DAS28-ESR = disease activity score 28 using erythrocyte sedimentation rate; HAQ = Health Assessment Questionnaire; SSc = Systemic sclerosis; Rodnan m = Rodnan modified; SpA = spondyloarthritis; BASDAI= Bath Ankylosing Spondylitis Disease Activity Index; BASFI= Bath Ankylosing Spondylitis Functional Index. SD = standard deviation; IQR = interquartile rate.

During the period of observation (mean time of 11.1 ± 8.9 years for RA, 9.8 ± 6.1 years for SLE, 10.4 ± 8.5 years for SpA and 8.8 ± 7.18 years for SSc) none of our patients developed thyroid cancer.

## DISCUSSION

Clustering of specific autoimmune diseases such as thyroiditis and systemic autoimmune diseases such as rheumatic diseases in the same patient is commonly noted,^8^ although the reasons for these associations are not completely clear. A common genetic background is one of the proposed explanations for this link.^8^ Another explanation that has been put forward is that there may have been exposure to a common triggering event such as a pollutant or a viral infection. In this regard, infections due to the Epstein-Barr virus^28^ and tobacco exposure^17^ have been implicated in both situations.

The results from the present study showed that SLE and SSc patients had greater quantities of antithyroid antibodies with or without hypothyroidism than did healthy controls. This higher prevalence had already been noted in several other studies, as detailed below.

Regarding SLE, a Colombian study^5^ found that the prevalence of hypothyroidism was 12% in their cohort, thus corroborating our findings. A meta-analysis by Pan et al.^29^ showed that their SLE patients were positive for Tg antibodies 2.9 times more frequently and for TPO antibodies 2.2 times frequently than healthy controls. In another study,^30^ it was observed that antithyroid antibody levels correlated with serum levels of other antibodies that are characteristic for SLE, such as anti-Sm, ribonucleoprotein (RNP) and dsDNA antibodies. However, those authors did not study the influence of presence of these antibodies on the clinical profile of the disease. Kumar et al.^31^ found that the presence of thyroid dysfunction was greater in patients with high degrees of disease activity, as measured using SLEDAI, while other studies had contrary findings.^32^^,^^33^ We did not find any association either with activity or with the cumulative harm index. However, the disease activity of our SLE patients was quite low, with median SLEDAI of zero.

One interesting finding from the present study, which had already been observed by Viggiano et al.,^34^ is that in SLE patients without hypothyroidism, anti-Tg antibodies were more common than anti-TPO antibodies. This outcome was unique to SLE patients, given that it was not found in the other rheumatic diseases studied here. Since anti-Tg antibodies are less specific for autoimmune thyroid disorder,^4^ it is possible to assume that its appearance may, at least partially, be due to unspecific polyclonal activation of B cells. Fluctuating levels of antithyroid antibodies over time have been reported in some SLE patients,^32^ thus validating this hypothesis. Nevertheless, it is possible that patients with persistently elevated antithyroid antibodies will progress to thyroid disease in the future, considering that these may be found in serum many years before the disease appears.^35^

A profile similar to SLE was found in SSc patients, with higher prevalence of thyroid autoantibodies with or without hypothyroidism than in controls. SSc patients showed a higher odds ratio for the presence of antithyroid antibodies. According to the literature, the percentage of antithyroid antibodies in SSc ranges from 12 to 52%^36^^,^^37^^,^^38^^,^^39^^,^^40^^,^^41^ and the percentage of hypothyroidism from 2.4 to 26%.^38^^,^^42^ A previous study showed that presence of hypothyroidism in SSc patients was associated with higher prevalence of pulmonary hypertension.^13^

It is important to highlight the high prevalence of thyroid autoimmunity in SLE and SSc cases in daily practice, since this is associated with higher prevalence of thyroid cancer.^43^^,^^44^ High frequency of papillary thyroid cancer in SSc patients in this context was noted by Antonelli et al.^44^ They hypothesized that this link could be due to the association of thyroid cancer with the chronic inflammatory process from thyroiditis or to a role played by a common proto-oncogene that activates the pathway of extracellular signal-regulated kinases (ERKs) found in SSc and in thyroid cancer. Tissue fibrosis (the hallmark of SSc) is also linked to increased rates of tumor appearance.^44^ Regarding SLE, findings from a meta-analysis by Zhang et al.^43^ that included seven studies revealed that the overall standardized incidence rate for thyroid cancer was 2.2 times higher than in controls. None of our SLE patients has developed thyroid cancer so far.

In the present study, we did not find that thyroid autoantibodies, either with or without thyroid dysfunction, were more common in either RA or SpA than in controls. A study on an Italian population showed high occurrence of thyroid autoantibodies with low prevalence of hormonal alterations.^45^ A meta-analysis on the prevalence of thyroid autoantibodies showed that there was increased prevalence of Tg and TPO antibodies, with OR of 3.1 and 2.3 respectively.^11^ Nevertheless, when a subgroup analysis according to patients’ geographical location was done, it showed that the American RA population did not have an increased rate of antithyroid antibodies, thus emphasizing the need for local studies. In addition, El Sheriff et al.^2^ found that thyroid disorders were significantly more common in SLE patients (50%) than in RA patients (15%).

Some authors have been unable to link thyroid autoantibodies with any specific clinical profile for RA.^35^ Others noted that RA patients who were positive for TPO antibodies showed greater progression of carotid intima media thickness, thus suggesting that this antibody may amplify the cardiovascular risk in this disease.^46^ However, another research group described thyroid autoantibodies as being more common in RA patients with high disease activity, as measured through DAS 28, anemia and seropositivity.^35^ We found a tendency towards an association for TPO antibodies and hypothyroidism with disease activity. However, judging disease activity in RA through instruments that use tender and swollen joint counts, such as DAS-28, may be misleading in this context. It has been reported that patients with autoimmune thyroid disease may have rheumatic manifestations even without glandular dysfunction.^2^ Arthralgia and even arthritis of small joints such as the wrist and hand are found in patients with thyroid autoimmunity without concomitant connective tissue disease.^7^^,^^35^ Punzi et al.^47^ observed antimicrosomal antibodies in the synovial fluid of patients one year prior to antibody detection in serum, and one year prior to the diagnosis of hypothyroidism. Another interesting observation is that RA patients with secondary fibromyalgia or chronic widespread pain had more TPOab than did those without this.^12^ It is possible to speculate that the articular component of thyroid autoimmunity may contribute towards increasing the painful symptoms.

SpA has been studied much less in this context. Contrary to our findings, Peluso et al.^16^ found that thyroid disorder was more common in SpA patients than in controls and they correlated this with occurrences of peripheral arthritis and long-duration disease. These authors did not report what influence drug treatment had on their sample, but others have noted that the frequency of thyroid disorder was lower in SpA patients receiving anti-TNF-alpha treatment.^48^ The same was observed in RA patients using adalimumab.^49^ This may at least partially explain the dissimilarity of these results. In our sample, 17.5% (14/80) of our SpA patients were on anti-TNF drugs. The different ethnic backgrounds of the samples studied may be another explanation.

Our study had a cross-sectional design, and this is one of its limitations. As already mentioned, patients with persistent autoantibody positivity, especially anti-TPO, may develop thyroid disease in the future.^35^ Also, the study design did not allow us to prove any causal association. Likewise, we were unable to determine what influence antirheumatic immunosuppressive drug treatment has on antibody positivity. Further studies, with longitudinal monitoring and higher numbers of patients are needed to answer these questions. On the other hand, it should be stressed that this study included a control group that was from the same geographical region as the patients, with similar exposure to iodine deficiency, and that all data were corrected for gender, age and tobacco exposure.

## CONCLUSION

It was found in our sample that SLE and SSc are diseases associated with higher prevalence of thyroid autoantibodies in patients with and without hypothyroidism. This was not seen in our SpA and RA samples. No link could be established between the presence of thyroid autoantibodies and the results from any of the instruments used to measure inflammatory activity and functional impairment in these cases of rheumatic diseases.
